# Asymptotic Degree Distributions in Random Threshold Graphs

**DOI:** 10.3390/e28060603

**Published:** 2026-05-27

**Authors:** Armand M. Makowski, Siddharth Pal

**Affiliations:** 1 Department of Electrical and Computer Engineering, University of Maryland, College Park, MD 20742, USA; 2 RTX BBN Technologies, Columbia, MD 21046, USA

**Keywords:** random graphs, degree distribution, asymptotic analysis, random threshold graphs

## Abstract

We discuss several limiting degree distributions for a class of homogeneous random graphs, known as random threshold graphs, in the many node regime. This analysis is carried out under a weak assumption on the distribution of the underlying fitness variable. This assumption, which is satisfied by the exponential distribution, determines a natural scaling under which the following limiting results are shown: the nodal degree distribution, i.e., the distribution of *any* node, converges in distribution to a limiting pmf. However, for each d=0,1,…, the *fraction* of nodes with degree *d* converges only in distribution to a *non-degenerate* random variable Π(d) (whose distribution depends on *d*), and *not* in probability to the aforementioned limiting nodal pmf as is customarily expected. The distribution of Π(d) is identified only through its characteristic function. Implications of this result include the following: (i) the empirical node distribution may not be used either as a proxy for or estimate of the limiting nodal pmf; (ii) even in homogeneous graphs, the network-wide degree distribution and the nodal degree distribution may capture vastly different information; and (iii) random threshold graphs with exponential distributed fitness do *not* provide an alternative scale-free model to the Barabási–Albert model as was argued by some authors; the two models cannot be meaningfully compared in terms of their degree distributions.

## 1. Introduction

Graphs as network models are routinely studied through their degree distributions, and much of the attention has focused on the *empirical* degree distribution that records the fractions of nodes with a given degree value. This distribution, which is easy to obtain from network measurements, has been found in many networks to obey a *power law* [1] (Section 1.4): If the network comprises a large number *n* of nodes and if there are $N_{n}\left(d\right)$ nodes with degree *d* among them, then the data reveals a behavior of the form

(1) $$\frac{N_{n}\left(d\right)}{n}≃Cd^{-\alpha }$$

for some α in the range [2,3] (although there are occasional exceptions) and C>0 [2,3]. See the monograph [1] (Section 4.2) for an introductory discussion and references, and the paper by Clauset et al. [4] for a principled statistical framework; see also the more recent paper by Voitalov et al. [5]. Statements such as (1) are usually left somewhat vague as the range for *d* is never carefully specified (in relation to *n*); networks where (1) was observed are often said to be *scale-free*.

The Barabási–Albert model came to prominence as the first random graph model to formally demonstrate the possibility of power law degree distribution in large networks [2]. The original Barabási–Albert model is a growth model which relies on the mechanism of *preferential attachment*—newly arriving nodes attach themselves to existing nodes with a probability proportional to their degrees at the time of arrival. As the number *n* of nodes increases, Bollobás et al. [6] proved that

(2) $$\frac{N_{n}\left(d\right)}{n}{\;\overset{P}{⟶}}_{n}\;p_{\mathrm{BA}}\left(d\right),\;d=0,1,\ldots$$

where $\left(\frac{N_{n}\left(d\right)}{n},\;d=0,1,\ldots \right)$ is the empirical degree distribution of the graph with *n* nodes, and the limiting pmf $p_{\mathrm{BA}}=\left(p_{\mathrm{BA}}\left(d\right),\;d=0,1,\ldots \right)$ on N has the power-tail behavior

(3) $$p_{\mathrm{BA}}\left(d\right)∼d^{-3}\;\left(d\to \infty \right).$$

Many generalizations of the Barabási–Albert model have been proposed over the years. Typically the convergence (2) still holds for some limiting pmf $p=\left(p(d),\;d=0,1,\ldots \right)$ on N with (3) replaced by $p\left(d\right)∼d^{-\tau }$ (d→∞) for some τ>0. The various models distinguish themselves from each other by their ability to achieve a value τ in a particular range [1] (Section 4.2).

Although in some contexts preferential attachment is a reasonable assumption, it is predicated on the degree of existing nodes being available to newly arriving nodes. There are many situations where this assumption is questionable, and where the creation of a link between two nodes may instead result from a mutual benefit based on their intrinsic attributes, e.g., authority, friendship, social success, wealth, etc. *Random threshold graph* models, which were proposed by Caldarelli et al. [7], incorporate this viewpoint in its simplest form as follows: let $\left\{\xi ,\xi_{k},\;k=1,2,\ldots \right\}$ denote i.i.d. $R_{+}$-valued random variables (rvs) with $\xi_{k}$ expressing the “fitness” level associated with node *k*. With *n* nodes and a threshold θ>0, the random threshold graph T(n,θ) postulates that two distinct nodes *i* and *j* form a connection (and there is an undirected edge between them) if

$$\xi_{i}+\xi_{j}>\theta ,\;\begin{array}{c} i\neq j\\ i,j=1,\ldots ,n. \end{array}$$

Interest in random threshold graphs has been spurred by the following observations. The distribution of the degree rvs $D_{n,1}\left(\theta \right),\ldots ,D_{n,n}\left(\theta \right)$ in T(n;θ) (defined formally by (11)) is the same for all nodes. It is therefore appropriate to speak of *the* degree distribution of a node in T(n;θ), namely that of $D_{n,1}\left(\theta \right)$. Now consider the case when the fitness variable ξ is exponentially distributed with parameter λ>0, and the threshold θ is scaled with the number *n* of nodes according to the scaling $\theta^{★}:N_{0}\to R_{+}$ given by

(4) $$\theta_{n}^{★}=\lambda^{-1}\log n,\;n=1,2,\ldots$$

In that setting, Fujihara et al. [8] have shown the distributional convergence

(5) $$D_{n,1}\left(\theta_{n}^{★}\right){⟹}_{n}D$$

where the limiting rv *D* has pmf $p_{\mathrm{Fuj}}=\left(p_{\mathrm{Fuj}}\left(d\right),\;d=0,1,\ldots \right)$ on N with power-tail behavior

(6) $$p_{\mathrm{Fuj}}\left(d\right)∼d^{-2}\;\left(d\to \infty \right).$$

The result (5)–(6) has led some researchers [7,9] to conclude that random threshold graphs can model scale-free networks (with τ=2) without having to resort to either a growth process or a preferential attachment mechanism, and as such they provide an *alternative* to the Barabási–Albert model. However, a moment of reflection should lead one to question this conclusion given the evidence available so far. Indeed, the statement (2) concerns an empirical degree distribution which is computed network-wide, whereas the convergence (5)–(6) addresses the distributional behavior of the degree of a *single* node, its distribution being identical across nodes.

A natural question is whether this discrepancy can be resolved in the large network limit. More precisely, for each d=0,1,…, let $N_{n}\left(d;\theta \right)$ denote the number of nodes in T(n;θ) which have degree *d*, namely

$$N_{n}\left(d;\theta \right)=\sum_{k=1}^{n}1\left[D_{n,k}\left(\theta \right)=d\right],\;n=2,3,\ldots$$

In analogy with (2), when the fitness variable ξ is exponentially distributed with parameter λ>0, it is certainly appropriate to wonder whether the convergence

(7) $$\frac{N_{n}\left(d;\theta_{n}^{★}\right)}{n}{\;\overset{P}{⟶}}_{n}\;p_{\mathrm{Fuj}}\left(d\right)$$

takes place with the pmf $p_{\mathrm{Fuj}}$ being the one appearing at (5)–(6). Only then would random threshold graphs (under exponentially distributed fitness) be confirmed as a *bona fide* scale-free alternative model to the Barabási–Albert model (as described by (2)–(3)).

In this paper, for each d=0,1,…, we show that there exists a *non-degenerate*[0,1]-valued rv Π(d) such that

(8) $$\frac{N_{n}\left(d;\theta_{n}^{★}\right)}{n}{⟹}_{n}\Pi \left(d\right)$$

where the scaling $\theta^{★}:N_{0}\to R_{+}$ is the one defined at (4). In fact, we establish such a result for a very large class of fitness distributions (with the scaling $\theta^{★}:N_{0}\to R_{+}$ modified accordingly). The non-degeneracy of the rv Π(d) in (8) implies that (7) *cannot* hold, and random threshold graphs with exponential distributed fitness do *not* provide an alternative scale-free model to the Barabási–Albert model (as understood by (2)). Only the convergence (2) has meaning in the preferential attachment model while the convergence (5) has no equivalent there, the situation being reversed for random threshold graphs. The two models cannot be meaningfully compared in terms of their degree distributions! Thus, *even* in homogeneous graphs, the network-wide degree distribution and the nodal degree distribution may capture vastly different information. This issue was also investigated more broadly by the authors in the references [10,11,12]; see comments following Corollary 1.

We close with a summary of the contents of the paper: Random threshold graphs are introduced in Section 2 together with the needed notation and assumptions. As we consider situations that generalize the case of exponentially distributed fitness rvs, the scaling (4) is now replaced by a scaling $\theta^{★}:N_{0}\to R_{+}$ satisfying Assumption 1. This assumption is determined by the probability distribution of ξ, and ensures the convergence $D_{n,1}\left(\theta_{n}^{★}\right){⟹}_{n}D$ for some limiting rv *D* which is conditionally Poisson (given ξ) [Proposition 1]. Section 3 presents the main result of the paper [Theorem 1]. In the setting of Section 2, under Assumption 1, the distributional convergence (8) holds with a non-degenerate limit identified only through its characteristic function (35)–(36). A proof of Theorem 1 is given in Section 5 and is rooted in the method of moments [via Proposition 3 established in Section 6]. The main technical step is contained in Proposition 2; a proof of this multi-dimensional version of Proposition 1 is presented in several steps discussed in Section 7, Section 8, Section 9 and Section 10. In Section 4, we illustrate through limited simulations the failure of (7) and the validity of (8) in the case of random threshold graphs with exponentially distributed fitness.

## 2. Random Threshold Graphs

First, we detail some notation and conventions: The random variables (rvs) under consideration are all defined on the same probability triple (Ω,F,P). The construction of a sufficiently large probability triple carrying all needed rvs is standard and omitted in the interest of brevity. All probabilistic statements are made with respect to the probability measure P, and we denote the corresponding expectation operator by E. The notation ${\;\overset{P}{⟶}}_{n}$ (resp. ${⟹}_{n}$) is used to signify convergence in probability (resp. convergence in distribution) (under P) with *n* going to infinity; see the monographs [13,14,15] for definitions and properties. If *E* is a subset of Ω, then $1\left[E\right]$ denotes the indicator of the set *E* with the usual understanding that $1\left[E\right]\left(\omega \right)=1$ (resp. $1\left[E\right]\left(\omega \right)=0$) if ω∈E (resp. ω∉E). The symbol N (resp. $N_{0}$) denotes the set of non-negative (resp. positive) integers, and we often write $R_{+}$ for [0,∞).

**The model**—The setting is that of [16]. Let $\left\{\xi ,\xi_{k},\;k=1,2,\ldots \right\}$ denote a collection of i.i.d. $R_{+}$-valued rvs defined on the probability triple (Ω,F,P), each distributed according to a given (probability) distribution function F:R→[0,1]. With ξ acting as a generic representative for this sequence of i.i.d. rvs, we have

$$P\left[\xi \leq x\right]=F\left(x\right),\;x\in R.$$

At minimum, we assume that *F* is a *continuous* function on R with support on $R_{+}$, namely

(9) F(x)=0,x≤0.

Once *F* is specified, random thresholds graphs are characterized by two parameters, namely the number *n* of nodes and a threshold value θ>0. The network comprises *n* nodes, labeled k=1,…,n, and to each node *k* we assign a *fitness* variable (or weight) $\xi_{k}$ For distinct k,ℓ=1,…,n, the nodes *k* and *ℓ* are declared to be adjacent if

(10) $$\xi_{k}+\xi_{\ell }>\theta ,$$

in which case we say that an undirected link exists between these two nodes. The *random threshold* graph T(n;θ) is the (undirected) random graph defined on the set of vertices {1,…,n} with adjacency notion (10).

For each k=1,2,…,n, the degree of node *k* in T(n;θ) is the rv $D_{n,k}\left(\theta \right)$ given by

(11) $$D_{n,k}\left(\theta \right)=\sum_{\ell =1,\;\ell \neq k}^{n}1\left[\xi_{k}+\xi_{\ell }>\theta \right].$$

Under the enforced independence assumptions, conditionally on $\xi_{k}$, the rv $D_{n,k}\left(\theta \right)$ is a Binomial rv $\mathrm{Bin}(n-1;1-F\left(\theta -\xi_{k}\right))$. The rvs $D_{n,1}\left(\theta \right),\ldots ,D_{n,n}\left(\theta \right)$ being exchangeable, let $D_{n}\left(\theta \right)$ denote any N-valued rv distributed according to their common pmf.

**Existence of a limiting degree distribution**—Throughout we make the following assumption on *F*.

**Assumption** **1.** *There exists a scaling $\theta^{★}:N_{0}\to R_{+}$ with the property $\lim_{n\to \infty }\theta_{n}^{★}=\infty$ such that*

(12) $$\lim_{n\to \infty }n\left(1-F(\theta_{n}^{★}-x)\right)=\lambda \left(x\right),\;x\geq 0$$

*for some non-identically zero mapping*$\lambda :R_{+}\to R_{+}$.

The mapping $\lambda :R_{+}\to R_{+}$ is necessarily non-decreasing. The following result overlaps with a similar result by Fujihara et al. [8] (Thm. 2, p. 362).

**Proposition** **1.** *Under Assumption 1, there exists an N-valued rv D such that*

(13) $$D_{n}\left(\theta_{n}^{★}\right){⟹}_{n}D.$$

*The rv D is conditionally Poisson (given ξ) with pmf given by*

(14) $$P\left[D=d\right]=E\left[\frac{\lambda {\left(\xi \right)}^{d}}{d!}\cdot e^{-\lambda (\xi )}\right],\;d=0,1,\ldots$$

The convergence (13) is equivalent to

(15) $$\lim_{n\to \infty }P\left[D_{n}\left(\theta_{n}^{★}\right)=d\right]=P\left[D=d\right],\;d=0,1,\ldots$$

If the mapping $\lambda :R_{+}\to R_{+}$ assumes a constant value c>0, i.e., λ(x)=c for all x≥0, then the rv *D* is a Poisson rv with parameter *c*.

**Proof.**  Fix n=2,3,…, θ>0 and *z* in R. Standard pre-conditioning arguments yield

$$\begin{array}{c} E\left[z^{D_{n}\left(\theta \right)}\right]=E\left[\prod_{\ell =2}^{n}z^{1\left[\xi_{1}+\xi_{\ell }>\theta \right]}\right]=E\left[{\left(E{\left[z^{1\left[x+\xi >\theta \right]}\right]}_{x=\xi_{1}}\right)}^{n-1}\right] \end{array}$$

under the enforced independence assumptions where we note that

$$\begin{array}{ccc} E\left[z^{1\left[x+\xi >\theta \right]}\right] & = & P\left[x+\xi \leq \theta \right]+P\left[x+\xi >\theta \right]z\\ & = & 1-\left(1-z\right)\left(1-F(\theta -x)\right),\;x\in R. \end{array}$$

Using (9), we get

(16) $$\begin{array}{ccc} E\left[z^{D_{n}\left(\theta \right)}\right] & = & E\left[{\left(1-\left(1-z\right)\left(1-F(\theta -\xi )\right)\right)}^{n-1}\right]\\ & = & z^{n-1}P\left[\xi >\theta \right]+E\left[1\left[\xi \leq \theta \right]{\left(1-\left(1-z\right)\left(1-F(\theta -\xi )\right)\right)}^{n-1}\right]. \end{array}$$

Replace θ by $\theta_{n}^{★}$ in (16) according to the scaling $\theta^{★}:N_{0}\to R_{+}$ stipulated in Assumption 1, and let *n* go to infinity in the resulting equality *when* |z|≤1. This results in both $\lim_{n\to \infty }P\left[\xi >\theta_{n}^{★}\right]z^{n-1}=0$ and $\lim_{n\to \infty }n\left(1-z\right)\left(1-F(\theta_{n}^{★}-\xi )\right)=\left(1-z\right)\lambda \left(\xi \right)$, and it is then well known [17] (Prop. 3.1.1., p. 116) that

$$\lim_{n\to \infty }1\left[\xi \leq \theta_{n}^{★}\right]{\left(1-\left(1-z\right)\left(1-F(\theta_{n}^{★}-\xi )\right)\right)}^{n-1}=e^{-(1-z)\lambda (\xi )}.$$

Invoking the Bounded Convergence Theorem, we obtain

$$\lim_{n\to \infty }E\left[z^{D_{n}\left(\theta_{n}^{★}\right)}\right]=E\left[e^{-(1-z)\lambda (\xi )}\right],\;\left|z\right|\leq 1$$

and the desired conclusion (13)–(14) follows as we note that the right-hand side is the probability generating function (pgf) of the pmf (14). □

Assumption 1 holds in a number of interesting cases; in what follows, we use the standard notation $x^{+}=\max\left(x,0\right)$ for *x* in R. When ξ is exponentially distributed with parameter λ>0, namely

(17) $$P\left[\xi >x\right]=e^{-\lambda x^{+}},\;x\in R,$$

Assumption 1 holds with

(18) $$\lambda \left(x\right)=e^{\lambda x},\;x\geq 0$$

if we take $\theta_{n}^{★}=\lambda^{-1}\log n$ for all n=1,2,…. In this case, the pmf of *D* is the pmf $p_{\mathrm{Fuj}}$ appearing at (5)–(6); it is given by

(19) $$p_{\mathrm{Fuj}}\left(d\right)=P\left[D=d\right]=E\left[\frac{{\left(e^{\lambda \xi }\right)}^{d}}{d!}\cdot e^{-e^{\lambda \xi }}\right]=\int_{0}^{\infty }\left(\frac{e^{dx}}{d!}\cdot e^{-e^{x}}\right)\cdot e^{-x}dx,\;d=0,1,\ldots$$

as we substitute (18) into the expression (14); note that the rv λξ is exponentially distributed with unit parameter if ξ is exponentially distributed with parameter λ.

The second case deals with heavy-tailed rvs. The rv ξ is said to be a Pareto rv with parameters ν>0 and a>0 if

(20) $$P\left[\xi >x\right]={\left(\frac{a}{a+x^{+}}\right)}^{\nu },\;x\in R.$$

Assumption 1 holds with $\theta_{n}^{★}=a\sqrt[{\nu }]{n}$ for all n=1,2,…, and λ(x)=1 for all x≥0, in which case *D* is a Poisson rv with unit parameter.

## 3. The Main Results

Fix n=2,3,… and θ>0. For each d=0,1,…, the rv $N_{n}\left(d;\theta \right)$ defined by

(21) $$N_{n}\left(d;\theta \right)=\sum_{k=1}^{n}1\left[D_{n,k}\left(\theta \right)=d\right]$$

counts the number of nodes which have degree *d* in T(n;θ), while the fraction of nodes with degree *d* in T(n;θ) is given by

(22) $$P_{n}\left(d;\theta \right)=\frac{N_{n}\left(d;\theta \right)}{n}.$$

The main result of the paper is concerned with the following distributional convergence.

**Theorem** **1.** *Assume Assumption 1 to hold. For each d=0,1,…, there exists a non-degenerate [0,1]-valued rv Π(d) such that*

(23) $$P_{n}\left(d;\theta_{n}^{★}\right){⟹}_{n}\Pi \left(d\right)$$

*where the scaling $\theta^{★}:N_{0}\to R_{+}$ is the one postulated in Assumption 1. Moreover, it holds that $E\left[\Pi (d)\right]=P\left[D=d\right]$ and Var[Π(d)]>0 with D being the limiting rv whose existence is established in Proposition 1*.

In the course of proving Theorem 1 in Section 5, we determine the distribution of the rv Π(d) through its characteristic function (36). The non-degeneracy of the rv Π(d) has the following immediate and important consequence.

**Corollary** **1.** *Assume Assumption 1 to hold. For each d=0,1,…, the sequence $\{P_{n}\left(d;\theta_{n}^{★}\right),$n=1,2,…} cannot converge in probability to a constant, i.e., there exists no constant L(d) such that*

(24) $$P_{n}\left(d;\theta_{n}^{★}\right){\;\overset{P}{⟶}}_{n}\;L\left(d\right).$$

Corollary 1 was announced in the conference paper [11] when the fitness variables are exponentially distributed; in [12] the failure of the convergence (24) was shown, also in the exponential case, with the help of asymptotic results for order statistics. Here, a fuller picture is obtained under the minimal Assumption 1: Corollary 1 is an immediate byproduct of the weak convergence (23) and of the non-degenerate nature of the limiting rv Π(d).

The remainder of the paper is concerned with establishing Theorem 1; its proof uses the method of moments, and builds on two technical results, Propositions 2 and 3, which are stated below. Proposition 2 is a multi-dimensional version of Proposition 1, and provides the core technical content behind Theorem 1.

**Proposition** **2.** *Assume Assumption 1 to hold. For each r=1,2,…, there exists an $N^{r}$-valued rv $\left(D_{1},\ldots ,D_{r}\right)$ such that*

(25) $$\left(D_{n,1}\left(\theta_{n}^{★}\right),\ldots ,D_{n,r}\left(\theta_{n}^{★}\right)\right){⟹}_{n}\left(D_{1},\ldots ,D_{r}\right).$$

*The limiting rvs $D_{1},\ldots ,D_{r}$ are exchangeable, but not independent, each being distributed according to the limiting rv D whose existence is established in Proposition 1*.

Proposition 3 stated next relies on the multi-dimensional convergence given in Proposition 2 and provides the convergence result needed to apply the method of moments.

**Proposition** **3.** *Assume Assumption 1 to hold. For each r=1,2,…, we have*

(26) $$\lim_{n\to \infty }E\left[P_{n}{\left(d;\theta_{n}^{★}\right)}^{r}\right]=P\left[D_{1}=d,\ldots ,D_{r}=d\right],\;d=0,1,\ldots$$

*where the $N^{r}$-valued rv $\left(D_{1},\ldots ,D_{r}\right)$ is the limiting rv whose existence was established in Proposition 2*.

As we are about to embark on establishing these results, we comment on the order of the presentation: Theorem 1 follows readily from Proposition 3 with a short proof given in Section 5. Proposition 3 is an easy byproduct of Proposition 2 and is established next in Section 6. Finally, we turn to proving Proposition 2 which contains the technical contents of the paper; a lengthy multi-step proof is provided in Section 7, Section 8, Section 9 and Section 10.

## 4. Simulation Results

Before turning to the proofs, we illustrate the difference between the convergence statements (2) and (8) through a limited set of simulation experiments which are discussed in this section. Throughout, the fitness variable ξ is taken to be exponentially distributed with parameter λ=1, and the threshold is scaled in accordance with (4), namely $\theta_{n}^{★}=\log n$ for each n=2,3,….

With the number *n* of nodes given, we generate *R* mutually independent versions of the random threshold graph $T(n;\theta_{n}^{★})$; these realizations are denoted $T^{\left(1\right)}\left(n;\theta_{n}^{★}\right),T^{\left(2\right)}\left(n;\theta_{n}^{★}\right),\ldots ,T^{\left(R\right)}\left(n;\theta_{n}^{★}\right)$. For each k=1,2,…,n and r=1,2,…,R, let $D_{n,k}^{\left(r\right)}\left(\theta_{n}^{★}\right)$ denote the degree of node *k* in the random graph $T^{\left(r\right)}\left(n;\theta_{n}^{★}\right)$, and for d=0,1,…, let $N_{n}^{\left(r\right)}\left(d;\theta_{n}^{★}\right)$ denote the number of nodes with degree *d* in $T^{\left(r\right)}\left(n;\theta_{n}^{★}\right)$.

**The rv Π(d) is non-degenerate**—Fix d=0,1,…. On the strength of Theorem 1, a natural way to produce an estimate for the probability distribution of the rv Π(d) is to follow a simple two-step procedure. On the basis of the *R* i.i.d. realizations of the random threshold graph $T(n;\theta_{n}^{★})$, a standard estimate of the probability distribution of $P_{n}\left(d;\theta_{n}^{★}\right)$ is provided by the histogram

$$H_{n,R}\left(d;x\right)=\frac{1}{R}\sum_{r=1}^{R}1\left[\frac{N_{n}^{\left(r\right)}\left(d;\theta_{n}^{★}\right)}{n}\leq x\right],\;x\in R.$$

The probability distribution $x\to H_{n,R}\left(d;x\right)$ has support on [0,1] with $H_{n,R}\left(d;x\right)=0$ for x<0 and $H_{n,R}\left(d;x\right)=1$ for 1≤x, conditions necessarily satisfied by the probability distribution of Π(d). Under the enforced independence assumptions, for *each* n=1,…, the Glivenko-Cantelli Theorem [13] (p. 103) asserts that

(27) $$\lim_{R\to \infty }\left(\underset{0\leq x\leq 1}{\sup}\left|H_{n,R}\left(d;x\right)-P\left[P_{n}\left(d;\theta_{n}^{★}\right)\leq x\right]\right|\right)=0\;\mathrm{a.s.}$$

Thus, for large *R* (possibly dependent on *n*), the probability distribution of the rv $P_{n}\left(d;\theta_{n}^{★}\right)$ is uniformly well approximated by the histogram $x\to H_{n,R}\left(d;x\right)$ with high probability.

On the other hand, Theorem 1 states that

(28) $$\lim_{n\to \infty }P\left[P_{n}\left(d;\theta_{n}^{★}\right)\leq x\right]=P\left[\Pi (d)\leq x\right],\;x\in C\left(\Pi \left(d\right)\right)$$

where C(Π(d)) is the set of points of continuity of the probability distribution of Π(d). Thus, for each *x* in C(Π(d)), the probability $P\left[\Pi (d)\leq x\right]$ will be well approximated by $P\left[P_{n}\left(d;\theta_{n}^{★}\right)\leq x\right]$ when *n* is large (possibly dependent on *x*).

Combining (27) and (28) with a simple triangle inequality argument naturally leads us to propose the approximation

(29) $$P\left[\Pi (d)\leq x\right]=\mathrm{Approx}H_{n,R}\left(d;x\right),\;x\in C\left(\Pi \left(d\right)\right)$$

with integers *n* and *R* selected sufficiently large. Put differently, we expect the probability distribution of Π(d) to be well approximated by the histogram $x\to H_{n,R}\left(d;x\right)$ if we select both *n* and *R* to be large. If such a histogram were found to be very different from a step function, this would provide compelling evidence that (24) cannot hold, and that the rv Π(d) is *not* degenerate.

**Figure 1:**
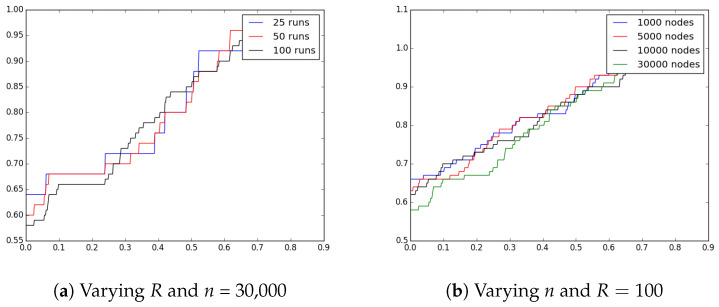
Histogram $H_{n,R}\left(d;.\right)$ for degree d=0 with varying number of nodes *n* and the number of runs *R* held fixed, and vice versa.

**Figure 2:**
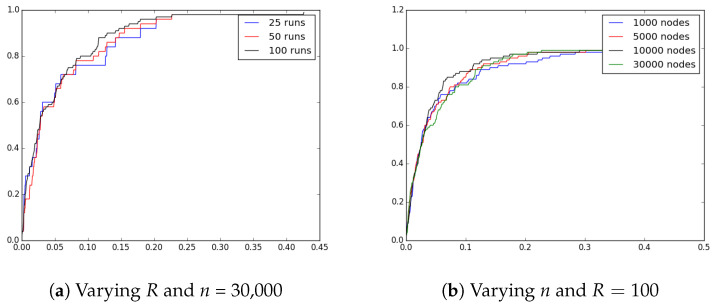
Histogram $H_{n,R}\left(d;.\right)$ for d=5 with varying number of nodes *n* and the number of runs *R* held fixed, and vice versa.

**Figure 3:**
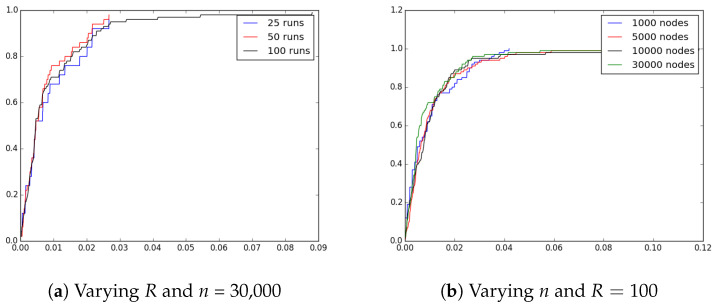
Histogram $H_{n,R}\left(d;.\right)$ for d=10 with varying number of nodes *n* and the number of runs *R* held fixed, and vice versa.

In Figure 1, Figure 2 and Figure 3, we show the approximating histogram $H_{n,R}\left(d;.\right)$ for the values d=0,5,10, with a varying number *R* of runs and a varying number *n* of graph sizes. Figure 1 deals with d=0. Figure 1a shows the histograms for *n* = 30,000 with increasing values R=25,50,100; the shape of the corresponding histograms do not change significantly. In Figure 1b, with R=100, increasing the graph size *n* = 1000, 5000,10,000, 30,000 also does not change the histograms significantly. This points to the non-degeneracy of Π(0) since in all cases the approximating histograms are reasonably close together but never approximate, even remotely, a step function. Figure 2 and Figure 3 exhibit histogram plots for d=5,10 under similar conditions; the conclusions are identical to the ones reached in the case d=0, with the evidence being possibly even stronger since the histograms appear to “bend” in a concave manner.

**Empirical degree distribution vs. nodal degree distribution**—As noted earlier, in the exponential case, the limiting rv *D* appearing at (13) has pmf $p_{\mathrm{Fuj}}$ given by (19), namely

(30) $$p_{\mathrm{Fuj}}\left(d\right)=P\left[D=d\right]=\int_{0}^{\infty }\left(\frac{e^{dx}}{d!}\cdot e^{-e^{x}}\right)\cdot e^{-x}dx,\;d=0,1,\ldots$$

For d=0, we numerically evaluate the appropriate integral with

$$p_{\mathrm{Fuj}}\left(0\right)=\int_{0}^{\infty }e^{-e^{x}}\cdot e^{-x}dx=\int_{0}^{1}e^{-\frac{1}{t}}dt≃0.1485.$$

For d=2,3,…, we note from (30) that

$$\begin{array}{ccc} p_{\mathrm{Fuj}}\left(d\right) & = & \frac{1}{d!}\int_{0}^{\infty }e^{(d-1)x}\cdot e^{-e^{x}}dx\\ & = & \frac{1}{d!}\int_{1}^{\infty }t^{d-2}\cdot e^{-t}dt\;\left[Change\;of\;variablet=e^{x}\right]\\ & = & \frac{1}{d!}\left(\int_{0}^{\infty }t^{d-2}\cdot e^{-t}dt-\int_{0}^{1}t^{d-2}\cdot e^{-t}dt\right)\\ & = & \frac{1}{d!}\left(\left(d-2\right)!-\int_{0}^{1}t^{d-2}\cdot e^{-t}dt\right), \end{array}$$

whence

$$p_{\mathrm{Fuj}}\left(d\right)-\frac{1}{d(d-1)}=\frac{\int_{0}^{1}t^{d-2}\cdot e^{-t}dt}{d!}.$$

The bound

$$\varepsilon \left(d\right)=\left|p_{\mathrm{Fuj}}\left(d\right)-\frac{1}{d(d-1)}\right|\leq \frac{1}{d!}$$

readily yields (6). This suggests the approximation

$$p_{\mathrm{Fuj}}\left(d\right)=\mathrm{Approx}\frac{1}{d(d-1)}$$

whose accuracy dramatically increases with *d* increasing as ε(d) decreases very rapidly, e.g., the approximations $p_{\mathrm{Fuj}}\left(5\right)=\mathrm{Approx}\frac{1}{20}$ (with an error less than 1/120) and $p_{\mathrm{Fuj}}\left(10\right)=\mathrm{Approx}\frac{1}{90}$ (with an error less than 1/10!) are already tight.

**Figure 4:**
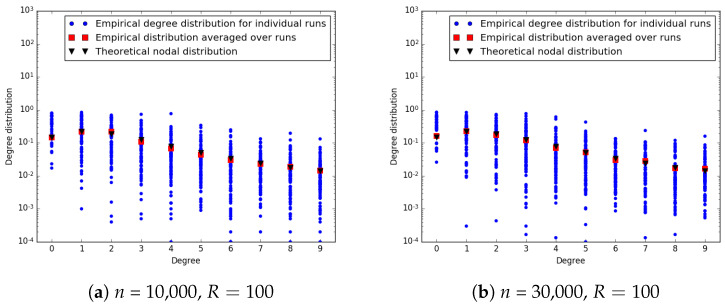
The nodal degree distribution $p_{\mathrm{Fuj}}$ was plotted against the empirical degree distribution $\frac{N_{n}^{\left(r\right)}\left(.;\theta_{n}^{★}\right)}{n}$ for various runs r=1,2,…,R.

Next, we explore the behavior of the empirical degree distribution (22) along the scaling (4) (with λ=1) as generated through a single network realization. We do so by plotting the histograms

(31) $$\frac{N_{n}^{\left(r\right)}\left(d;\theta_{n}^{★}\right)}{n}=\frac{1}{n}\sum_{k=1}^{n}1\left[D_{n,k}^{\left(r\right)}\left(\theta_{n}^{★}\right)=d\right],\;\begin{array}{c} d=0,1,\ldots \\ r=1,\ldots ,R \end{array}$$

for various values of *d* and *r*, and large *n*, and comparing against the corresponding value for $p_{\mathrm{Fuj}}\left(d\right)$. In Figure 4, we plot the histogram $\frac{N_{n}^{\left(r\right)}\left(.;\theta_{n}^{★}\right)}{n}$ for different runs r=1,2,…,R and varying graph sizes *n* = 10,000, 30,000, and observe high variability with respect to the nodal degree distribution $p_{\mathrm{Fuj}}$, which does not change as the graph size is increased.

One might be tempted to smooth out the variability observed in Figure 4 by averaging the empirical degree distributions (31) over the *R* i.i.d. realizations $T^{\left(1\right)}\left(n;\theta_{n}^{★}\right),T^{\left(2\right)}\left(n;\theta_{n}^{★}\right),\ldots ,T^{\left(R\right)}\left(n;\theta_{n}^{★}\right)$, resulting in the statistic

(32) $$\frac{1}{R}\sum_{r=1}^{R}\frac{N_{n}^{\left(r\right)}\left(d;\theta_{n}^{★}\right)}{n},\;d=0,1,\ldots .$$

Fix d=0,1,…. Under these circumstances, the Strong Law of Large Numbers yields

(33) $$\begin{array}{ccc} \lim_{R\to \infty }\frac{1}{R}\sum_{r=1}^{R}\frac{N_{n}^{\left(r\right)}\left(d;\theta_{n}^{★}\right)}{n} & = & E\left[\frac{N_{n}\left(d;\theta_{n}^{★}\right)}{n}\right]\;\mathrm{a.s.} \end{array}$$

with

$$E\left[\frac{N_{n}\left(d;\theta_{n}^{★}\right)}{n}\right]=E\left[\frac{1}{n}\sum_{k=1}^{n}1\left[D_{n,k}\left(\theta_{n}^{★}\right)=d\right]\right]=P\left[D_{n}\left(\theta_{n}^{★}\right)=d\right]$$

by exchangeability. On the other hand, we have $\lim_{n\to \infty }P\left[D_{n}\left(\theta_{n}^{★}\right)=d\right]=p_{\mathrm{Fuj}}\left(d\right)$ by virtue of Proposition 1. Combining these observations yields the approximation

(34) $$\frac{1}{R}\sum_{r=1}^{R}\frac{N_{n}^{\left(r\right)}\left(d;\theta_{n}^{★}\right)}{n}=\mathrm{Approx}p_{\mathrm{Fuj}}\left(d\right)$$

for large *n* and *R*. The goodness of the approximation (34) is noted in Figure 4, where the empirical distribution averaged over R=100 runs is observed to be very close to the nodal degree distribution. However, the accuracy of the approximation (34) does in no way imply the validity of (7). In fact, the mistaken belief that (7) holds, implicitly assumed in the papers [7,9], might have stemmed from using the smoothed estimate provided by the averaging (34).

## 5. A Proof of Theorem 1

Fix d=0,1,…. We establish the weak convergence of the sequence $\left\{P_{n}\left(d;\theta_{n}^{★}\right),\;n=2,3,\ldots \right\}$ by arguments based on the method of moments; for details concerning this approach, see the references [14] (Thm. 4.5.5, p. 99) and [18] (Thm. 6.1, p. 140).

Proposition 3 suggests considering the mapping $\Phi_{d}:R\to C$ given by

(35) $$\Phi_{d}\left(t\right)=1+\sum_{r=1}^{\infty }\frac{{\left(it\right)}^{r}}{r!}\cdot P\left[D_{1}=d,\ldots ,D_{r}=d\right],\;t\in R.$$

This definition is well posed with $\Phi_{d}\left(t\right)$ always an element of C since

$$1+\sum_{r=1}^{\infty }\frac{{\left|t\right|}^{r}}{r!}\cdot P\left[D_{1}=d,\ldots ,D_{r}=d\right]\leq \sum_{r=0}^{\infty }\frac{{\left|t\right|}^{r}}{r!}=e^{\left|t\right|},\;t\in R.$$

In particular, the mapping $\Phi_{d}:R\to C$ is analytic on R, and hence continuous at t=0. However, at this point in the proof, it is not yet known whether $\Phi_{d}$ is the characteristic function of a rv.

We close that gap as follows: For each n=2,3,…, let $\Phi_{d,n}:R\to C$ denote the characteristic function of the rv $P_{n}\left(d;\theta_{n}^{★}\right)$, i.e.,

$$\Phi_{d,n}\left(t\right)=E\left[e^{itP_{n}\left(d;\theta_{n}^{★}\right)}\right],\;t\in R.$$

The obvious bound $0\leq P_{n}\left(d;\theta_{n}^{★}\right)\leq 1$ implies the uniform bounds

$$\left|1+\sum_{r=1}^{R}\frac{{\left(it\right)}^{r}}{r!}\cdot P_{n}{\left(d;\theta_{n}^{★}\right)}^{r}\right|\leq 1+\sum_{r=1}^{\infty }\frac{{\left|t\right|}^{r}}{r!}\cdot P_{n}{\left(d;\theta_{n}^{★}\right)}^{r}\leq \sum_{r=0}^{\infty }\frac{{\left|t\right|}^{r}}{r!}=e^{\left|t\right|},\;\begin{array}{c} t\in R,\\ R=1,2,\ldots \end{array}$$

Therefore, applying the Bounded Convergence Theorem (with *R* going to infinity), separately to the real and imaginary parts, we readily validate the series expansion

$$\Phi_{d,n}\left(t\right)=1+\sum_{r=1}^{\infty }\frac{{\left(it\right)}^{r}}{r!}\cdot E\left[P_{n}{\left(d;\theta_{n}^{★}\right)}^{r}\right],\;t\in R.$$

For each *t* in R, it now follows that

$$\begin{array}{ccc} \Phi_{d,n}\left(t\right)-\Phi_{d}\left(t\right) & = & \sum_{r=1}^{\infty }\frac{{\left(it\right)}^{r}}{r!}\cdot \left(E\left[{\left(P_{n}\left(d;\theta_{n}^{★}\right)\right)}^{r}\right]-P\left[D_{1}=d,\ldots ,D_{r}=d\right]\right). \end{array}$$

Picking a positive integer *R*, we get

$$\begin{array}{c} \left|\Phi_{d,n}\left(t\right)-\Phi_{d}\left(t\right)\right|\leq \sum_{r=1}^{R}\frac{{\left|t\right|}^{r}}{r!}\cdot \left|E\left[P_{n}{\left(d;\theta_{n}^{★}\right)}^{r}\right]-P\left[D_{1}=d,\ldots ,D_{r}=d\right]\right|+2\sum_{r=R+1}^{\infty }\frac{{\left|t\right|}^{r}}{r!}. \end{array}$$

For each ε>0, there exists a positive integer $R^{★}\left(\varepsilon ,t\right)$ (independent of *n*) such that

$$\sum_{r=R+1}^{\infty }\frac{{\left|t\right|}^{r}}{r!}\leq \varepsilon ,\;R\geq R^{★}\left(\varepsilon ,t\right),$$

and on that range, we obtain

$$\begin{array}{c} \underset{n\to \infty }{lim sup}\left|\Phi_{d,n}\left(t\right)-\Phi_{d}\left(t\right)\right|\\ \;\;\;\;\leq \sum_{r=1}^{R}\frac{{\left|t\right|}^{r}}{r!}\cdot \underset{n\to \infty }{lim sup}\left(\left|E\left[P_{n}{\left(d;\theta_{n}^{★}\right)}^{r}\right]-P\left[D_{1}=d,\ldots ,D_{r}=d\right]\right|\right)+2\varepsilon \end{array}$$

by the usual arguments. Using Proposition 3, we readily conclude that ${lim sup}_{n\to \infty }\left|\Phi_{d,n}\left(t\right)-\Phi_{d}\left(t\right)\right|\leq 2\varepsilon$, whence $\lim_{n\to \infty }\Phi_{d,n}\left(t\right)=\Phi_{d}\left(t\right)$ since ε>0 is arbitrary.

By a standard result due to Cramér and Lévy [14] (Thm. 6.3.2, p. 161) [15] (Thm. 1, p. 320), the mapping $\Phi_{d}:R\to C$, being continuous at t=0 with $\Phi_{d}\left(0\right)=1$, must be the characteristic function of some rv, say Π(d), namely

(36) $$E\left[e^{it\Pi (d)}\right]=\Phi_{d}\left(t\right),\;t\in R$$

and the sequence $\left\{P_{n}\left(d;\theta_{n}^{★}\right),\;n=2,3,\ldots \right\}$ converges weakly to the rv Π(d).

For each d=0,1,…, we read from (35) and (36) that $E\left[\Pi (d)\right]=P\left[D_{1}=d\right]$ and $E\left[\Pi {\left(d\right)}^{2}\right]=P\left[D_{1}=d,D_{2}=d\right]$; hence,

(37) $$\begin{array}{ccc} \mathrm{Var}\left[\Pi (d)\right] & = & P\left[D_{1}=d,D_{2}=d\right]-P\left[D_{1}=d\right]\cdot P\left[D_{2}=d\right]\\ & = & \mathrm{Cov}\left[1\left[D_{1}=d\right]\cdot 1\left[D_{2}=d\right]\right]. \end{array}$$

The strict inequality $\mathrm{Var}\left[\Pi (d)\right]>0$ can be seen from the discussion at the end of Section 7. See also the references [10,12] for a discussion in the special case when the fitness rv is exponentially distributed. ■

## 6. A Proof of Proposition 3

Fix θ>0, d=0,1,… and r=1,2,…, and pick n>r; there is no loss of generality in doing so, here and elsewhere, as we are concerned with *n* large asymptotics. Let $P_{n,r}$ denote the collection of all *ordered* arrangements of *r distinct* elements drawn from the set {1,…,n}. Any such arrangement can be identified with a one-to-one mapping π:{1,…,r}→{1,…,n}.

We begin with the well-known identity

(38) $$\begin{array}{c} \prod_{s=0}^{r-1}\left(N_{n}\left(d;\theta \right)-s\right)=\sum_{\pi \in P_{n,r}}\left(\prod_{t=1}^{r}1\left[D_{n,\pi (t)}\left(\theta \right)=d\right]\right). \end{array}$$

Taking expectations on both sides of (38), we obtain

(39) $$\begin{array}{ccc} E\left[\prod_{s=0}^{r-1}\left(N_{n}\left(d;\theta \right)-s\right)\right] & = & \sum_{\pi \in P_{n,r}}E\left[\prod_{t=1}^{r}1\left[D_{n,\pi (t)}\left(\theta \right)=d\right]\right]\\ & = & \left|P_{n,r}\right|\cdot P\left[D_{n,1}\left(\theta \right)=d,\ldots ,D_{n,r}\left(\theta \right)=d\right] \end{array}$$

by the exchangeability of the rvs $D_{n,1}\left(\theta \right),\ldots ,D_{n,n}\left(\theta \right)$. Dividing both sides of (39) by $n^{r}$ leads to

(40) $$\begin{array}{c} E\left[\prod_{s=0}^{r-1}\left(P_{n}\left(d;\theta \right)-\frac{s}{n}\right)\right]=\frac{\left|P_{n,r}\right|}{n^{r}}\cdot P\left[D_{n,1}\left(\theta \right)=d,\ldots ,D_{n,r}\left(\theta \right)=d\right] \end{array}$$

with $\left|P_{n,r}\right|=n\left(n-1\right)\ldots \left(n-r+1\right)$.

Replace θ by $\theta_{n}^{★}$ in (40) according to the scaling $\theta^{★}:N_{0}\to R_{+}$ postulated in Assumption 1, and let *n* go to infinity in the resulting relation. We have $\lim_{n\to \infty }n^{-r}\left|P_{n,r}\right|=1$ by direct inspection while Proposition 2 yields

$$\lim_{n\to \infty }P\left[D_{n,1}\left(\theta_{n}^{★}\right)=d,\ldots ,D_{n,r}\left(\theta_{n}^{★}\right)=d\right]=P\left[D_{1}=d,\ldots ,D_{r}=d\right]$$

where the rvs $D_{1},\ldots ,D_{r}$ are the limiting rvs appearing in the convergence (25). This shows that

(41) $$\lim_{n\to \infty }E\left[\prod_{s=0}^{r-1}\left(P_{n}\left(d;\theta_{n}^{★}\right)-\frac{s}{n}\right)\right]=P\left[D_{1}=d,\ldots ,D_{r}=d\right].$$

Next, we readily check (say by recursion on *r*) that

$$\left|\prod_{s=0}^{r-1}\left(P_{n}\left(d;\theta_{n}^{★}\right)-\frac{s}{n}\right)-P_{n}{\left(d;\theta_{n}^{★}\right)}^{r}\right|\leq \sum_{s=0}^{r-1}\frac{s}{n}=\frac{r(r-1)}{2n},\;n=r,r+1,\ldots$$

and it immediately follows that

$$\lim_{n\to \infty }E\left[\left|\prod_{s=0}^{r-1}\left(P_{n}\left(d;\theta_{n}^{★}\right)-\frac{s}{n}\right)-P_{n}{\left(d;\theta_{n}^{★}\right)}^{r}\right|\right]=0.$$

Combining this last fact with the convergence (41), we obtain (26) by standard arguments, and the proof of Proposition 3 is now complete. ■

## 7. A Proof of Proposition 2

The remainder of the paper is devoted to the proof of Proposition 2. First, some notation: For each r=1,2,…, let $\xi_{r|1},\ldots ,\xi_{r|r}$ denote the values of the fitness rvs $\xi_{1},\ldots ,\xi_{r}$ arranged in increasing order, namely $\xi_{r|1}\leq \ldots \leq \xi_{r|r}$, with a lexicographic tiebreaker when needed. The rvs $\xi_{r|1},\ldots ,\xi_{r|r}$ are known as the *order statistics* associated with the collection $\xi_{1},\ldots ,\xi_{r}$; in particular, the rvs $\xi_{r|1}$ and $\xi_{r|r}$ are the minimum and maximum of the rvs $\xi_{1},\ldots ,\xi_{r}$, respectively [19]. In what follows, the permutation $\alpha_{r}:\left\{1,\ldots ,r\right\}\to \left\{1,\ldots ,r\right\}$ arranges the rvs $\xi_{1},\ldots ,\xi_{r}$ in increasing order, i.e.,

$$\xi_{r|s}=\xi_{\alpha_{r}\left(s\right)},\;s=1,\ldots ,r$$

(under the lexicographic tiebreaker). The permutation $\alpha_{r}$ being determined by the i.i.d. rvs $\xi_{1},\ldots ,\xi_{r}$, it is a random permutation which is *uniformly* distributed over the group $S_{r}$ of permutations of {1,…,r}. With the notation introduced earlier, write

(42) $$G_{r}\left(z_{1},\ldots ,z_{r}\right)=E\left[e^{-\sum_{t=1}^{r}\left(1-z_{\alpha_{r}\left(t\right)}\right)\left(\prod_{s=t+1}^{r}z_{\alpha_{r}\left(s\right)}\right)\lambda \left(\xi_{r|t}\right)}\right],\;\begin{array}{c} 0\leq z_{s}\leq 1,\\ \;s=1,\ldots ,r. \end{array}$$

By convention, the product of an empty set of factors is set to unity in the expression (42) and elsewhere in the discussion below.

Proposition 2 is an easy consequence of the following key analytical result whose proof is broken up in several steps discussed in Section 8, Section 9 and Section 10.

**Proposition** **4.** *Assume Assumption 1 to hold. For each r=1,2,…, we have*

(43) $$\begin{array}{c} \lim_{n\to \infty }E\left[\prod_{s=1}^{r}z_{s}^{D_{n,s}\left(\theta_{n}^{★}\right)}\right]=G_{r}\left(z_{1},\ldots ,z_{r}\right),\;\begin{array}{c} 0\leq z_{s}\leq 1\\ s=1,\ldots ,r \end{array} \end{array}$$

We devote the remainder of this section to show that Proposition 4 indeed implies Proposition 2.

**Convergence**—First, by the Bounded Convergence Theorem, we get

$$\lim_{z_{s}↑1,\;s=1,\ldots ,r}G_{r}\left(z_{1},\ldots ,z_{r}\right)=1$$

and the mapping $G_{r}:{\left[0,1\right]}^{r}\to R$ is therefore continuous at the point (1,…,1). This fact, coupled with the convergence (43), implies that $G_{r}$ is an *r*-dimensional pgf. Thus, there exists an $N^{r}$-valued rv, denoted $\left(D_{1},\ldots ,D_{r}\right)$, such that

(44) $$E\left[\prod_{s=1}^{r}z_{s}^{D_{s}}\right]=G_{r}\left(z_{1},\ldots ,z_{r}\right),\;\begin{array}{c} 0\leq z_{s}\leq 1,\\ \;s=1,\ldots ,r \end{array}$$

and the convergence (25) follows in the usual manner. ■

**Exchangeability in the limit**—To establish exchangeability, we proceed as follows: For each n=2,3,…, the rvs $D_{n,1}\left(\theta_{n}^{★}\right),\ldots ,D_{n,r}\left(\theta_{n}^{★}\right)$ are obviously exchangeable rvs, and the exchangeability of the limiting rvs $D_{1},\ldots ,D_{r}$ follows because exchangeability is preserved under the weak convergence (25). This fact could also be gleaned directly from (44) as we note from (42) that the mapping $G_{r}:{\left[0,1\right]}^{r}\to R$ is permutation-invariant in the sense that

$$G_{r}\left(z_{\sigma (1)},\ldots ,z_{\sigma (r)}\right)=G_{r}\left(z_{1},\ldots ,z_{r}\right),\;\begin{array}{c} 0\leq z_{s}\leq 1,\\ \;s=1,\ldots ,r \end{array}$$

for every permutation σ of the index set {1,…,r}: Indeed, the random permutation $\sigma \circ \alpha_{r}:\left\{1,\ldots ,r\right\}\to \left\{1,\ldots ,r\right\}:s\to \sigma \left(\alpha_{r}\left(s\right)\right)$ is uniform over $S_{r}$ since the random permutation $\alpha_{r}:\left\{1,\ldots ,r\right\}\to \left\{1,\ldots ,r\right\}$ is itself uniform over $S_{r}$, and the rvs $\xi_{1},\ldots ,\xi_{r}$ are i.i.d. rvs; details are left to the interested reader.

As expected, we retrieve Proposition 1 by looking at the case r=1, namely

(45) $$E\left[z_{i}^{D_{i}}\right]=E\left[e^{-\left(1-z_{i}\right)\lambda \left(\xi_{i}\right)}\right],\;\begin{array}{c} 0\leq z_{i}\leq 1\\ i=1,2. \end{array}$$

For r=2, we also find

(46) $$E\left[z_{1}^{D_{1}}z_{2}^{D_{2}}\right]=E\left[e^{-\left(1-z_{\alpha_{2}\left(1\right)}\right)z_{\alpha_{2}\left(2\right)}\lambda \left(\xi_{2|1}\right)-\left(1-z_{\alpha_{2}\left(2\right)}\right)\lambda \left(\xi_{2|2}\right)}\right],\;0\leq z_{1},z_{2}\leq 1.$$

Comparing (45) and (46), we can check that

(47) $$E\left[z_{1}^{D_{1}}z_{2}^{D_{2}}\right]\neq E\left[z_{1}^{D_{1}}\right]E\left[z_{2}^{D_{2}}\right],\;0\leq z_{1},z_{2}\leq 1$$

and the rvs $D_{1},\ldots ,D_{r}$ are therefore *not* independent. This completes the proof of Proposition 2.

To further illustrate this last point, consider the special case when the mapping $\lambda :R_{+}\to R_{+}$ appearing in Assumption 1 is constant, say λ(x)=c>0 for all x≥0, as would be the case for the Pareto distribution (20). The expressions (45) and (46) now become

$$E\left[z_{i}^{D_{i}}\right]=e^{-(1-z_{i})c}\;\begin{array}{c} 0\leq z_{i}\leq 1\\ i=1,2 \end{array}$$

and

$$E\left[z_{1}^{D_{1}}z_{2}^{D_{2}}\right]=e^{-(1-z_{1}z_{2})c},\;0\leq z_{1},z_{2}\leq 1.$$

Thus, each of the rvs $D_{1},\ldots ,D_{r}$ is Poisson distributed with parameter *c* and $P\left[D_{1}=D_{2}\right]=1$ since

$$P\left[D_{1}=d,D_{2}=d^{\prime }\right]=\frac{c^{d}}{d!}\cdot e^{-c}\cdot \delta \left(d,d^{\prime }\right),\;d,d^{\prime }=0,1,\ldots$$

Exchangeability yields $P\left[D_{1}=D_{j}\right]=1$ for every j=2,…,r; hence, $P\left[D_{1}=\ldots =D_{r}\right]=1$, and the rvs $D_{1},\ldots ,D_{r}$ are certainly not independent! Moreover, for each d=0,1,…, we get $P\left[D_{1}=d,\ldots ,D_{r}=d\right]=P\left[D_{1}=d\right]$ for all r=1,2,…, whence

(48) $$\begin{array}{ccc} \Phi_{d}\left(t\right) & = & 1+\sum_{r=1}^{\infty }\frac{{\left(it\right)}^{r}}{r!}\cdot P\left[D_{1}=d\right]\\ & = & 1+\left(e^{it}-1\right)\cdot P\left[D_{1}=d\right]\\ & = & \left(1-P\left[D_{1}=d\right]\right)+e^{it}\cdot P\left[D_{1}=d\right],\;t\in R. \end{array}$$

In other words, the distribution of the rv Π(d) is the two-point mass distribution $\left(1-P\left[D_{1}=d\right],P\left[D_{1}=d\right]\right)$ on the set {0,1} with $P\left[D_{1}=d\right]=\frac{c^{d}}{d!}\cdot e^{-c}$. Obviously,

$$\mathrm{Var}\left[\Pi \left(d\right)\right]=P\left[D_{1}=d\right]-P\left[D_{1}=d\right]\cdot P\left[D_{1}=d\right]=P\left[D_{1}=d\right]\cdot \left(1-P\left[D_{1}=d\right]\right)>0$$

since $0<P\left[D_{1}=d\right]<1$.

When the fitness rv ξ is exponentially distributed, explicit expressions were obtained for Var[Π(d)] by direct arguments in the earlier references [10,11,12]. ■

## 8. A Proof of Proposition 4—A Reduction Step

Throughout this section, the integer r=1,2,… and the parameter θ>0 are held fixed, and pick n>r. For each k=1,…,r, we write

$$D_{n,k}\left(\theta \right)=\sum_{\ell =1,\;\ell \neq k}^{r}1\left[\xi_{k}+\xi_{\ell }>\theta \right]+D_{n,k}^{\left(r\right)}\left(\theta \right)$$

with

$$D_{n,k}^{\left(r\right)}\left(\theta \right)=\sum_{\ell =r+1}^{n}1\left[\xi_{k}+\xi_{\ell }>\theta \right].$$

As the scaling $\theta^{★}:N_{0}\to R_{+}$ satisfies $\lim_{n\to \infty }\theta_{n}^{★}=\infty$, it is plain that

$$\lim_{n\to \infty }\max\left(\sum_{\ell =1,\;\ell \neq k}^{r}1\left[\xi_{k}+\xi_{\ell }>\theta_{n}^{★}\right],\;k=1,\ldots ,r\right)=0\;a.s.$$

and the desired convergence (43) takes place if and only if

(49) $$\lim_{n\to \infty }E\left[\prod_{s=1}^{r}z_{s}^{D_{n,s}^{\left(r\right)}\left(\theta_{n}^{★}\right)}\right]=G_{r}\left(z_{1},\ldots ,z_{r}\right),\;\begin{array}{c} 0\leq z_{s}\leq 1\\ s=1,\ldots ,r. \end{array}$$

Our first step towards establishing (49) is to evaluate the joint pgfs. Pick $z_{1},\ldots ,z_{r}$ in R. Under the enforced independence assumptions, it is plain that

(50) $$\begin{array}{ccc} E\left[\prod_{s=1}^{r}z_{s}^{D_{n,s}^{\left(r\right)}\left(\theta \right)}\right] & = & E\left[\prod_{s=1}^{r}z_{s}^{\sum_{\ell =r+1}^{n}1\left[\xi_{s}+\xi_{\ell }>\theta \right]}\right]\\ & = & E\left[\prod_{s=1}^{r}\prod_{\ell =r+1}^{n}z_{s}^{1\left[\xi_{s}+\xi_{\ell }>\theta \right]}\right]\\ & = & E\left[\prod_{\ell =r+1}^{n}\prod_{s=1}^{r}z_{s}^{1\left[\xi_{s}+\xi_{\ell }>\theta \right]}\right]\\ & = & E\left[E\left[\prod_{\ell =r+1}^{n}\prod_{s=1}^{r}z_{s}^{1\left[\xi_{s}+\xi_{\ell }>\theta \right]}|\xi_{1},\ldots ,\xi_{r}\right]\right]\\ & = & E\left[E{\left[\prod_{\ell =r+1}^{n}\prod_{s=1}^{r}z_{s}^{1\left[x_{s}+\xi_{\ell }>\theta \right]}\right]}_{x_{1}=\xi_{1},\ldots ,x_{r}=\xi_{r}}\right]. \end{array}$$

With arbitrary $x_{1},\ldots ,x_{r}$ in $R_{+}$, the rvs $\xi_{r+1},\ldots ,\xi_{n}$ being i.i.d. rvs, we get

$$\begin{array}{ccc} E\left[\prod_{\ell =r+1}^{n}\prod_{s=1}^{r}z_{s}^{1\left[x_{s}+\xi_{\ell }>\theta \right]}\right] & = & \prod_{\ell =r+1}^{n}E\left[\prod_{s=1}^{r}z_{s}^{1\left[x_{s}+\xi_{\ell }>\theta \right]}\right]\\ & = & F_{r}{\left(\theta ;z_{1},\ldots ,z_{r};x_{1},\ldots ,x_{r}\right)}^{n-r} \end{array}$$

upon setting

(51) $$\begin{array}{ccc} F_{r}\left(\theta ;z_{1},\ldots ,z_{r};x_{1},\ldots ,x_{r}\right) & = & E\left[\prod_{s=1}^{r}z_{s}^{1\left[x_{s}+\xi >\theta \right]}\right]\\ & = & E\left[\prod_{s=1}^{r}\left(1\left[x_{s}+\xi >\theta \right]z_{s}+1\left[x_{s}+\xi \leq \theta \right]\right)\right]\\ & = & E\left[\prod_{s=1}^{r}\left(1-\left(1-z_{s}\right)1\left[x_{s}+\xi >\theta \right]\right)\right]. \end{array}$$

Substituting back into (50), we conclude that

(52) $$E\left[\prod_{s=1}^{r}z_{s}^{D_{n,s}^{\left(r\right)}\left(\theta \right)}\right]=E\left[F_{r}{\left(\theta ;z_{1},\ldots ,z_{r};\xi_{1},\ldots ,\xi_{r}\right)}^{n-r}\right],$$

and the desired result (49) will hold if we show the convergence

(53) $$\lim_{n\to \infty }E\left[F_{r}{\left(\theta_{n}^{★};z_{1},\ldots ,z_{r};\xi_{1},\ldots ,\xi_{r}\right)}^{n-r}\right]=G_{r}\left(z_{1},\ldots ,z_{r}\right)$$

on the range

(54) $$0\leq z_{s}\leq 1,\;s=1,\ldots ,r.$$

■

## 9. A Proof of Proposition 4—A Decomposition

Fix θ>0 and r=1,2,…. To further analyze the expression (52) with $x_{1},\ldots ,x_{r}$ in $R_{+}$, we introduce the set of indices

$$S\left(\theta ;x_{1},\ldots ,x_{r}\right)=\left\{s=1,\ldots ,r:\;x_{s}>\theta \right\}.$$

There are two possibilities which we now explore in turn. Either $S(\theta ;x_{1},\ldots ,x_{r})$ is empty or it is not, leading to a natural decomposition expressed through Lemmas 1 and 2, respectively.

**Lemma** **1.** *With $x_{1},\ldots ,x_{r}$ in $R_{+}$, whenever $S(\theta ;x_{1},\ldots ,x_{r})$ is non-empty, we have*

(55) $$\begin{array}{c} F_{r}\left(\theta ;z_{1},\ldots ,z_{r};x_{1},\ldots ,x_{r}\right)\\ \;\;=\left(\prod_{s\in S(\theta ;x_{1},\ldots ,x_{r})}z_{s}\right)\cdot E\left[\prod_{s\notin S(\theta ;x_{1},\ldots ,x_{r})}^{r}\left(1-\left(1-z_{s}\right)1\left[x_{s}+\xi >\theta \right]\right)\right] \end{array}$$

*for all $z_{1},\ldots ,z_{r}$ in R*.

**Proof.**  Pick arbitrary $x_{1},\ldots ,x_{r}$ in $R_{+}$ such that $S(\theta ;x_{1},\ldots ,x_{r})$ is non-empty. For all $z_{1},\ldots ,z_{r}$ in R, it is easy to check by direct inspection from the expression (51) that (55) holds since $1-\left(1-z_{s}\right)1\left[x_{s}+\xi >\theta \right]=z_{s}$ whenever *s* belongs to $S(\theta ;x_{1},\ldots ,x_{r})$. □

As an immediate consequence of (55), we have the bounds

(56) $$\begin{array}{c} 0\leq F_{r}\left(\theta ;z_{1},\ldots ,z_{r};x_{1},\ldots ,x_{r}\right)\leq 1,\;\begin{array}{c} x_{1},\ldots ,x_{r}\in R_{+}\\ \mathrm{with}\\ |S(\theta ;x_{1},\ldots ,x_{r})|>0 \end{array} \end{array}$$

on the range (54), since there we have $0\leq 1-\left(1-z_{s}\right)1\left[x_{s}+\xi >\theta \right]\leq 1$ for all s=1,…,r.

We now turn to the case when the index set $S(\theta ;x_{1},\ldots ,x_{r})$ is empty, a fact characterized by the conditions

(57) $$x_{s}\leq \theta ,\;s=1,\ldots ,r.$$

It will be convenient to arrange the values $x_{1},\ldots ,x_{r}$ in increasing order, say $x_{\left(r|1\right)}\leq x_{\left(r|2\right)}\leq \ldots \leq x_{\left(r|r\right)}$, with a lexicographic tiebreaker. Let $a_{r}$ be any permutation of {1,…,r} such that $x_{\left(r|s\right)}=x_{a_{r}\left(s\right)}$ for all s=1,…,r. Obviously, this permutation is determined by the values $x_{1},\ldots ,x_{r}$. In what follows, we shall use the convention $x_{\left(r|0\right)}=-\infty$ and $x_{\left(r|r+1\right)}=\infty$.

**Lemma** **2.** *With $x_{1},\ldots ,x_{r}$ in $R_{+}$, whenever $S(\theta ;x_{1},\ldots ,x_{r})$ is empty, we have*

(58) $$\begin{array}{c} F_{r}\left(\theta ;z_{1},\ldots ,z_{r};x_{1},\ldots ,x_{r}\right)=\sum_{t=0}^{r}\left(\prod_{s=t+1}^{r}z_{a_{r}\left(s\right)}\right)\cdot \left(F\left(\theta -x_{\left(r|t\right)}\right)-F\left(\theta -x_{\left(r|t+1\right)}\right)\right) \end{array}$$

*for all $z_{1},\ldots ,z_{r}$ in R*.

**Proof.**  In what follows, the values $z_{1},\ldots ,z_{r}$ in R are held fixed. Given $x_{1},\ldots ,x_{r}$ in $R_{+}$ and θ>0, we define the events

$$A_{r|t}\left(x_{1},\ldots ,x_{r};\theta \right)=\left[x_{\left(r|t\right)}+\xi \leq \theta <x_{\left(r|t+1\right)}+\xi \right],\;t=0,1,\ldots ,r.$$

Under the enforced conventions, we have $A_{r|0}\left(x_{1},\ldots ,x_{r};\theta \right)=\left[\theta <x_{\left(r|1\right)}+\xi \right]$ and $A_{r|r}\left(x_{1},\ldots ,x_{r};\theta \right)=\left[x_{\left(r|r\right)}+\xi \leq \theta \right]$. When $S(\theta ;x_{1},\ldots ,x_{r})$ is empty, the r+1 events $A_{r|0}\left(x_{1},\ldots ,x_{r};\theta \right),\ldots ,A_{r|r}\left(x_{1},\ldots ,x_{r};\theta \right)$ are pairwise disjoint and form a partition of the sample space. Using this fact in the expression (51), we find

(59) $$\begin{array}{c} F_{r}\left(\theta ;z_{1},\ldots ,z_{r};x_{1},\ldots ,x_{r}\right)\\ \;\;\;=\sum_{t=0}^{r}E\left[1\left[A_{r|t}\left(x_{1},\ldots ,x_{r};\theta \right)\right]\prod_{s=1}^{r}\left(1-\left(1-z_{s}\right)1\left[x_{s}+\xi >\theta \right]\right)\right]. \end{array}$$

Three cases need to be considered.

(i) On the event $A_{r|0}\left(x_{1},\ldots ,x_{r};\theta \right)$, we have $\theta <x_{\left(r|1\right)}+\xi$, and thus $\theta <x_{s}+\xi$ for all s=1,…,r, so that

$$\prod_{s=1}^{r}\left(1-\left(1-z_{s}\right)1\left[x_{s}+\xi >\theta \right]\right)=\prod_{s=1}^{r}z_{s},$$

whence

(60) $$\begin{array}{c} E\left[1\left[A_{r|0}\left(x_{1},\ldots ,x_{r};\theta \right)\right]\prod_{s=1}^{r}\left(1-\left(1-z_{s}\right)1\left[x_{s}+\xi >\theta \right]\right)\right]\\ \;\;\;=\left(\prod_{s=1}^{r}z_{s}\right)\cdot P\left[\theta <x_{\left(r|1\right)}+\xi \right]\\ \;\;\;=\left(\prod_{s=1}^{r}z_{s}\right)\cdot \left(1-F(\theta -x_{\left(r|1\right)})\right). \end{array}$$

(ii) With t=1,…,r−1, on the event $A_{r|t}\left(x_{1},\ldots ,x_{r};\theta \right)$, we have the inequalities $x_{\left(r|1\right)}+\xi \leq \theta ,\ldots ,x_{\left(r|t\right)}+\xi \leq \theta$ and $\theta <x_{\left(r|t+1\right)}+\xi ,\ldots ,\theta <x_{\left(r|r\right)}+\xi$, whence

$$\prod_{s=1}^{r}\left(1-\left(1-z_{s}\right)1\left[x_{s}+\xi >\theta \right]\right)=\left(\prod_{s=t+1}^{r}z_{a_{r}\left(s\right)}\right).$$

We readily conclude that

(61) $$\begin{array}{c} E\left[1\left[A_{r|t}\left(x_{1},\ldots ,x_{r};\theta \right)\right]\prod_{s=1}^{r}\left(1-\left(1-z_{s}\right)1\left[x_{s}+\xi >\theta \right]\right)\right]\\ \;\;\;=\left(\prod_{s=t+1}^{r}z_{a_{r}\left(s\right)}\right)\cdot P\left[x_{\left(r|t\right)}+\xi \leq \theta <x_{\left(r|t+1\right)}+\xi \right]\\ \;\;\;=\left(\prod_{s=t+1}^{r}z_{a_{r}\left(s\right)}\right)\cdot \left(F\left(\theta -x_{\left(r|t\right)}\right)-F\left(\theta -x_{\left(r|t+1\right)}\right)\right). \end{array}$$

(iii) Finally, on the event $A_{r|r}\left(x_{1},\ldots ,x_{r};\theta \right)$, we have $x_{\left(r|r\right)}+\xi \leq \theta$, and thus $x_{s}+\xi \leq \theta$ for all s=1,…,r, so that

$$\prod_{s=1}^{r}\left(1-\left(1-z_{s}\right)1\left[x_{s}+\xi >\theta \right]\right)=1,$$

whence

(62) $$\begin{array}{ccc} E\left[1\left[A_{r|r}\left(x_{1},\ldots ,x_{r};\theta \right)\right]\prod_{s=1}^{r}\left(1-\left(1-z_{s}\right)1\left[x_{s}+\xi >\theta \right]\right)\right] & = & P\left[x_{\left(r|r\right)}+\xi \leq \theta \right]\\ & = & F(\theta -x_{\left(r|r\right)}). \end{array}$$

To complete the proof of (58), we substitute (60), (61) and (62) into (59), and recall that $F(\theta -x_{\left(r|0\right)})=1$ and $F(\theta -x_{\left(r|r+1\right)})=0$ under the conventions adopted here. □

## 10. A Proof of Proposition 4—Taking the Limit

In order to establish the convergence (49), we return to the expression (52) for the joint pgf of the relevant rvs.

**A useful intermediary fact**—Pick r=1,2,… and fix n=2,3,… with r<n. For arbitrary θ>0, consider $x_{1},\ldots ,x_{r}$ in $R_{+}$ and $z_{1},\ldots ,z_{r}$ in R. In what follows, it will be convenient to define

$$\Lambda_{r}\left(\theta ;z_{1},\ldots ,z_{r};x_{1},\ldots ,x_{r}\right)=1-F_{r}\left(\theta ;z_{1},\ldots ,z_{r};x_{1},\ldots ,x_{r}\right)$$

so that

$$F_{r}\left(\theta ;z_{1},\ldots ,z_{r};x_{1},\ldots ,x_{r}\right)=1-\Lambda_{r}\left(\theta ;z_{1},\ldots ,z_{r};x_{1},\ldots ,x_{r}\right).$$

Whenever $S(\theta ;x_{1},\ldots ,x_{r})$ is empty, Lemma 2 gives

$$\begin{array}{c} \Lambda_{r}\left(\theta ;z_{1},\ldots ,z_{r};x_{1},\ldots ,x_{r}\right) \end{array}$$

(63) $$\begin{array}{c} \;\;\;=1-\sum_{t=0}^{r}\left(\prod_{s=t+1}^{r}z_{a_{r}\left(s\right)}\right)\cdot \left(F\left(\theta -x_{\left(r|t\right)}\right)-F\left(\theta -x_{\left(r|t+1\right)}\right)\right) \end{array}$$

(64) $$\begin{array}{c} \;\;\;=-\sum_{t=0}^{r-1}\left(\prod_{s=t+1}^{r}z_{a_{r}\left(s\right)}\right)\cdot \left(F\left(\theta -x_{\left(r|t\right)}\right)-F\left(\theta -x_{\left(r|t+1\right)}\right)\right)+\left(1-F(\theta -x_{\left(r|r\right)})\right). \end{array}$$

Replace θ by $\theta_{n}^{★}$ in () according to the scaling $\theta^{★}:N_{0}\to R_{+}$ stipulated in Assumption 1. Letting *n* go to infinity in the resulting relation, we get

$$\lim_{n\to \infty }n\left(1-F(\theta_{n}^{★}-x_{\left(r|r\right)})\right)=\lambda \left(x_{\left(r|r\right)}\right)$$

and

(65) $$\begin{array}{c} \lim_{n\to \infty }n\left(F\left(\theta_{n}^{★}-x_{\left(r|t\right)}\right)-F\left(\theta_{n}^{★}-x_{\left(r|t+1\right)}\right)\right)\\ \;\;\;=\lim_{n\to \infty }n\left(\left(1-F(\theta_{n}^{★}-x_{\left(r|t+1\right)}\right)-\left(1-F(\theta_{n}^{★}-x_{\left(r|t\right)})\right)\right)\\ \;\;\;=\left\{\begin{array}{cc} \lambda (x_{\left(r|1\right)}) & \mathrm{if}\;t=0\\ \lambda \left(x_{\left(r|t+1\right)}\right)-\lambda \left(x_{\left(r|t\right)}\right) & \mathrm{if}\;t=1,\ldots ,r-1. \end{array}\right. \end{array}$$

As a result, with $S(\theta ;x_{1},\ldots ,x_{r})$ empty, we have

(66) $$\begin{array}{c} \lim_{n\to \infty }n\Lambda_{r}\left(\theta_{n}^{★};z_{1},\ldots ,z_{r};x_{1},\ldots ,x_{r}\right)\\ \;\;\;=-\lambda \left(x_{\left(r|1\right)}\right)\left(\prod_{s=1}^{r}z_{s}\right)-\sum_{t=1}^{r-1}\left(\prod_{s=t+1}^{r}z_{a_{r}\left(s\right)}\right)\left(\lambda \left(x_{\left(r|t+1\right)}\right)-\lambda \left(x_{\left(r|t\right)}\right)\right)+\lambda \left(x_{\left(r|r\right)}\right)\\ \;\;\;=-\sum_{t=1}^{r}\lambda \left(x_{\left(r|t\right)}\right)\left(\prod_{s=t}^{r}z_{a_{r}\left(s\right)}-\prod_{s=t+1}^{r}z_{a_{r}\left(s\right)}\right)\\ \;\;\;=\sum_{t=1}^{r}\lambda \left(x_{\left(r|t\right)}\right)\left(1-z_{a_{r}\left(t\right)}\right)\left(\prod_{s=t+1}^{r}z_{a_{r}\left(s\right)}\right), \end{array}$$

and the conclusion

$$\begin{array}{ccc} \lim_{n\to \infty }F_{r}{\left(\theta_{n}^{★};z_{1},\ldots ,z_{r};x_{1},\ldots ,x_{r}\right)}^{n-r} & = & \lim_{n\to \infty }{\left(1-\Lambda_{r}\left(\theta_{n}^{★};z_{1},\ldots ,z_{r};x_{1},\ldots ,x_{r}\right)\right)}^{n-r}\\ & = & e^{-\sum_{t=1}^{r}\lambda \left(x_{\left(r|t\right)}\right)\left(1-z_{a_{r}\left(t\right)}\right)\prod_{s=t+1}^{r}z_{a_{r}\left(s\right)}} \end{array}$$

follows by standard arguments [17] (Prop. 3.1.1., p. 116). ■

**In the limit**—Pick scalars $z_{1},\ldots ,z_{r}$ in the range (54). For each n=2,3,… with r<n, we start with the decomposition

(67) $$\begin{array}{c} E\left[F_{r}{\left(\theta_{n}^{★};z_{1},\ldots ,z_{r};\xi_{1},\ldots ,\xi_{r}\right)}^{n-r}\right]\\ \;\;\;=E\left[1\left[|S(\theta_{n}^{★};\xi_{1},\ldots ,\xi_{r})|>0\right]F_{r}{\left(\theta_{n}^{★};z_{1},\ldots ,z_{r};\xi_{1},\ldots ,\xi_{r}\right)}^{n-r}\right]\\ \;\;\;\;\;\;\;\;\;+E\left[1\left[|S(\theta_{n}^{★};\xi_{1},\ldots ,\xi_{r})|=0\right]F_{r}{\left(\theta_{n}^{★};z_{1},\ldots ,z_{r};\xi_{1},\ldots ,\xi_{r}\right)}^{n-r}\right]. \end{array}$$

The condition $\lim_{n\to \infty }\theta_{n}^{★}=\infty$ yields

$$\lim_{n\to \infty }1\left[|S(\theta_{n}^{★};\xi_{1},\ldots ,\xi_{r})|=0\right]=\lim_{n\to \infty }1\left[\xi_{1}\leq \theta_{n}^{★},\ldots ,\xi_{r}\leq \theta_{n}^{★}\right]=1,$$

whence $\lim_{n\to \infty }P\left[|S(\theta_{n}^{★};\xi_{1},\ldots ,\xi_{r})|=0\right]=1$, and the inequality (56) now implies

(68) $$\lim_{n\to \infty }E\left[1\left[|S(\theta_{n}^{★};\xi_{1},\ldots ,\xi_{r})|>0\right]F_{r}{\left(\theta_{n}^{★};z_{1},\ldots ,z_{r};\xi_{1},\ldots ,\xi_{r}\right)}^{n-r}\right]=0$$

on the range (54).

Next, for each n=1,2,… such that r<n, we also have

(69) $$\begin{array}{c} E\left[1\left[|S(\theta_{n}^{★};\xi_{1},\ldots ,\xi_{r})|=0\right]F_{r}{\left(\theta_{n}^{★};z_{1},\ldots ,z_{r};\xi_{1},\ldots ,\xi_{r}\right)}^{n-r}\right]\\ \;\;\;=E\left[1\left[|S(\theta_{n}^{★};\xi_{1},\ldots ,\xi_{r})|=0\right]{\left(1-\Lambda_{r}\left(\theta_{n}^{★};z_{1},\ldots ,z_{r};\xi_{1},\ldots ,\xi_{r}\right)\right)}^{n-r}\right] \end{array}$$

with (63) yielding

(70) $$\begin{array}{c} \Lambda_{r}\left(\theta_{n}^{★};z_{1},\ldots ,z_{r};\xi_{1},\ldots ,\xi_{r}\right)\\ \;\;\;=1-F\left(\theta_{n}^{★}-\xi_{r|r}\right)-\sum_{t=0}^{r-1}\left(\prod_{s=t+1}^{r}z_{\alpha_{r}\left(s\right)}\right)\cdot \left(F\left(\theta_{n}^{★}-\xi_{r|t}\right)-F\left(\theta_{n}^{★}-\xi_{r|t+1}\right)\right). \end{array}$$

As we pass from (63) to (70), we recall that the order statistics $\xi_{r|1},\ldots ,\xi_{r|r}$ associated with $\xi_{1},\ldots ,\xi_{r}$ were introduced in the statement of Proposition 4, together with the *random* permutation $\alpha_{r}:\left\{1,\ldots ,r\right\}\to \left\{1,\ldots ,r\right\}$. The random permutation $\alpha_{r}$ coincides with the deterministic permutation $a_{r}$ induced by the values $x_{1},\ldots ,x_{r}$ with $x_{1}=\xi_{1},\ldots ,x_{r}=\xi_{r}$.

On the range (54), it is plain that

$$0\leq \left(\prod_{s=t+1}^{r}z_{\alpha_{r}\left(s\right)}\right)\leq 1,\;t=0,\ldots ,r-1,$$

while the bounds $0\leq \Lambda_{r}\left(\theta_{n}^{★};z_{1},\ldots ,z_{r};\xi_{1},\ldots ,\xi_{r}\right)\leq 1$ hold by direct inspection of (70), whence $\left|F_{r}{\left(\theta_{n}^{★};z_{1},\ldots ,z_{r};\xi_{1},\ldots ,\xi_{r}\right)}^{n-r}\right|\leq 1$. With the fact $\lim_{n\to \infty }1\left[|S(\theta_{n}^{★};\xi_{1},\ldots ,\xi_{r})|=0\right]$=1 noted earlier, we see that the convergence

$$\begin{array}{c} \lim_{n\to \infty }1\left[|S(\theta_{n}^{★};\xi_{1},\ldots ,\xi_{r})|=0\right]F_{r}{\left(\theta_{n}^{★};z_{1},\ldots ,z_{r};\xi_{1},\ldots ,\xi_{r}\right)}^{n-r}\\ \;\;\;=e^{-\sum_{t=1}^{r}\lambda \left(\xi_{r|t}\right)\left(1-z_{\alpha_{r}\left(t\right)}\right)\prod_{s=t+1}^{r}z_{\alpha_{r}\left(s\right)}} \end{array}$$

takes place boundedly, and the Bounded Convergence Theorem can then be applied to yield

(71) $$\begin{array}{c} \lim_{n\to \infty }E\left[1\left[|S(\theta_{n}^{★};\xi_{1},\ldots ,\xi_{r})|=0\right]F_{r}{\left(\theta_{n}^{★};z_{1},\ldots ,z_{r};\xi_{1},\ldots ,\xi_{r}\right)}^{n-r}\right]\\ \;\;\;=E\left[e^{-\sum_{t=1}^{r}\lambda \left(\xi_{r|t}\right)\left(1-z_{\alpha (t)}\right)\prod_{s=t+1}^{r}z_{\alpha_{r}\left(s\right)}}\right]\\ \;\;\;=G_{r}\left(z_{1},\ldots ,z_{r}\right). \end{array}$$

Let *n* go to infinity in (67). Collecting (68) and (71), we conclude that (53) indeed holds on the range (54) as desired. ■

## Data Availability

The original contributions presented in this study are included in the article. Further inquiries can be directed to the corresponding author.
